# Creation and Initial Validation of the Skin Dysmorphia Scale: Time for a New Concept to Arise in the Medical Field

**DOI:** 10.1111/jocd.70647

**Published:** 2026-01-05

**Authors:** Feten Fekih‐Romdhane, Rabih Hallit, Marita Hakim, Sahar Obeid, Diana Malaeb, Fouad Sakr, Mariam Dabbous, Frederic Harb, Souheil Hallit

**Affiliations:** ^1^ The Tunisian Center of Early Intervention in Psychosis, Department of Psychiatry “Ibn Omrane” Razi Hospital Manouba Tunisia; ^2^ Tunis El Manar University, Faculty of Medicine of Tunis Tunis Tunisia; ^3^ School of Medicine and Medical Sciences Holy Spirit University of Kaslik Jounieh Lebanon; ^4^ Department of Infectious Disease Notre Dame des Secours University Hospital Center Byblos Lebanon; ^5^ Department of Infectious Disease Bellevue Medical Center Mansourieh Lebanon; ^6^ Social and Education Sciences Department, School of Arts and Sciences Lebanese American University Byblos Lebanon; ^7^ College of Pharmacy Gulf Medical University Ajman United Arab Emirates; ^8^ School of Pharmacy Lebanese International University Beirut Lebanon; ^9^ Department of Biomedical Sciences, Faculty of Medicine and Medical Sciences University of Balamand Koura Lebanon; ^10^ Applied Science Research Center Applied Science Private University Amman Jordan

**Keywords:** body dysmorphic disorder, psychometric properties, scale development, skin dysmorphia

## Abstract

**Background:**

Skin dysmorphia is an emerging construct that reflects experiences of concern with perceived imperfections pertaining to skin coupled with an obsession with skincare routines to achieve flawless skin. It increasingly poses unique challenges to healthcare professionals and thus urgently necessitates a comprehensive approach to assessment and management. This study represents the first concerted effort to design and validate a psychometrically sound scale for use in clinical assessment and future research on skin dysmorphia that we called “Skin Dysmorphia Scale” (SDS).

**Method:**

A cross‐sectional survey was performed in July–August 2025 in Lebanon among adults from the general population.

**Results:**

After removal of 11 items with significant cross‐loadings, seven items remained which loaded onto a single factor and resulted in high internal consistency reliability (Cronbach's alpha = 0.83). A positive, moderate correlation was found between skin dysmorphia tendencies and general body dysmorphia symptoms, thereby supporting the convergent validity of the SDS. Heavier TikTok users showed significantly higher skin dysmorphia tendencies. Statistically significant correlations were observed between SDS scores and higher depression‐anxiety symptoms. Moreover, skin dysmorphia symptoms were significantly and inversely correlated with self‐esteem levels.

**Conclusion:**

Preliminary analyses suggest that the newly developed SDS is a valid and reliable instrument for assessing skin dysmorphic concern and ensuring adequate, timely intervention or referral. We believe that it is timely and useful that skin dysmorphia be given high priority and be formally recognized by the medical and scientific community so that affected individuals can get the necessary medical or mental health care. Clinicians and researchers are encouraged to begin using the SDS in their practice.

## 1 Introduction

Body Dysmorphic Disorder (BDD) is a mental health condition in which the affected individual experiences preoccupation with one or more perceived flaws or defects in physical appearance that are only slightly or not at all observable to others [1]. BDD often results in feelings of embarrassment and shame and leads to hours spent in mirror gazing in attempts of reassurance about physical appearance. Fear of negative criticism can lead the individual to social avoidance and isolation, absence from school or loss of work. In addition, individuals with BDD are more likely to meet criteria for an anxiety‐related or depressive disorder and exhibit high suicidality [2]. Although different parts of the body could be a source of preoccupation, one of the most commonly involved is skin [3]. A systematic review with meta‐analysis found that BDD happens in 13% of dermatology patients [4]. Another systematic review indicated that up to 3.2% in the general population and 21% of people in general dermatology cohorts suffer from BDD [5]. Individuals with skin‐related dysmorphic concerns face potential dangers, including pursuing unnecessary or excessive cosmetic procedures or surgery [6], thus generating substantial costs to the healthcare system [7].

Different terminologies have been utilized to define and describe individuals with obsessive preoccupation with perceived skin flaws, including dysmorphobia, dermatological non‐disease, dermatological hypochondriasis, dysmorphic syndrome [8], and skin dysmorphology [9]. In the present work, the terminology “skin dysmorphia” will be used, by analogy to the term “muscle dysmorphia” which refers to another specific subtype of BDD that focuses on muscularity [10]. Skin dysmorphia is therefore a form of body dysmorphia where the area of preoccupation is skin. It specifically depicts one's concern with perceived imperfections pertaining to skin, coupled with an obsession with skincare routines to achieve flawless skin, and which can consequently lead to impaired daily functioning and/or significant distress.

### 1.1 Skin Dysmorphia: Why Is It Important to Recognize This Entity?

Over the past years, the internet and social media have played a key role in the development and manifestation of BDD and skin dysmorphia [11]. Young people are increasingly exposed to viral beauty trends and persuasive marketing of skincare and cosmetic products through social media platforms, with more and more exposure to beauty regimens at an unprecedentedly young age. The unrealistic beauty standards in social media, the use of facial filters that create a smooth and lighten face (such as those found on TikTok), and the viewing of edited, idealized photos of influencers and celebrities have driven significant concerns with skin appearance and created extraordinary expectations [12]. Therefore, the rising preoccupation with perceived defects in the appearance of skin is driven by emerging cultural phenomena, including the societal pressure to have ageless skin and the unattainable beauty promises perpetuated by the global beauty industry. The desire to achieve perfect skin leads to adherence to strict skincare regimens, thus prioritizing immediate aesthetic appeal over long‐term wellbeing [13]. New trends encourage eating particular aliments or avoiding certain types of food to support skin health [14]. More particularly, and among all social media platforms, TikTok has grown in popularity within the past years and has become one of the most popular [12]. TikTok has created an influential environment that has been the catalyst for making young people wanting an ageless, glassy and glowy skin without any flaw, and has made them obsessed over trending beauty products [15, 16]. Research reported that TikTok content substantially influences the perceived ideal of how skin should look and has been usually the initial and reliable source of information about skincare products [15, 17]. However, while the existing empirical evidence reported a close connection between excessive social media use (any platforms) and (general) BDD [18, 19, 20], no previous studies have specifically focused on the relationship between TikTok use and skin dysmorphia.

Being obsessed with flawless skin and excessive preoccupation with skincare routines expose the affected person to major health risks, including irritation, scarring, inflammation, increased sun damage, along with negative psychological consequences [21]. For instance, there have been case reports that raised concerns about adverse effects experienced after trying beauty trends endorsed by social media influencers [22]. Other researchers reported a case of seizures and liver toxicity hepatitis following obsessive skincare habits in a previously healthy female [23]. Qualitative research has shown that having the skincare routine done and being satisfied with one's own skin were closely tied to feelings of “force,” “wellness,” “happiness,” “fulfillment,” and “self‐esteem.” In contrast, being dissatisfied with one's skin often relates to feelings of distress [14]. In the face of this new scourge, it is urgently needed to generate theoretical insights into and new empirical evidence about the characteristics and behaviors of skin dysmorphia.

### 1.2 Why Is a New Instrument Needed?

Despite its high prevalence and severity, BDD remains commonly undiagnosed by both psychiatrists and non‐psychiatric physicians, misunderstood and under‐researched [6, 24]. This could be due to lack of suspicion on clinical grounds, as symptoms can be overlapping with other mental health conditions or confusing to differentiate from non‐pathological dissatisfaction with body image [25]. Several self‐report measures exist to assess BDD, such as the Body Image Disturbance Questionnaire [26], the Body Dysmorphic Disorder Examination Self‐Report [27], the Body Dysmorphic disorder dimensional scale [28], the Body Dysmorphic Disorder Examination [29], the Dysmorphic Concern Questionnaire (DCQ) [30], and the Body Dysmorphic Disorder Questionnaire [31].

Although these measures demonstrated high specificity, sensitivity, reliability, and efficiency in screening for BDD, they may not capture the full range of symptoms that a person can experience [32]. In particular, none of the BDD measurements available were designed to specifically capture skin dysmorphia [32]. In addition, most of the existing instruments are generally outdated, as they were created and validated between 1994 and 2013. Therefore, symptoms and behaviors that have emerged in a rapidly evolving global landscape, under the influence of technological development and digitization, may not be represented. With the growing influence of social and digital media in shaping body image perception, several researchers called for the need of novel BDD instruments that take into consideration social media‐related behaviors [33], such as trending skincare routines [13].

### 1.3 Study's Objectives and Hypotheses

The lack of an accurate screening instrument for skin dysmorphia stands in significant contrast to its high prevalence and potential negative impact on the affected individual. Therefore, a logical next step appears to be the development of a validated tool to precisely evaluate the construct of skin dysmorphia. This study represents the first concerted effort to design and validate a psychometrically sound scale for use in clinical assessment and future research on skin dysmorphia that we called “Skin Dysmorphia Scale” (SDS). Specifically, our objectives were to operationalize skin dysmorphia and to establish the validity and reliability of the SDS. Exploratory then Confirmatory Factor Analysis will be run to explore the underlying factor structure based on an initial pool of items. Measurement invariance across sex will be verified. Convergent validity will be tested by investigating associations between the SDS and a conceptually similar measure (the DCQ). Concurrent validity will be determined through testing the correlations between the SDS and measures of TikTok addiction, depression, anxiety, and self‐esteem.

## 2 Method

### 2.1 Procedure, Sample, and Design

A cross‐sectional survey was performed in July–August 2025 in Lebanon. Our target participants were community adults aged 18 years and over, with Arabic reading and comprehension skills, who had access to the internet and who voluntarily accepted to take part in our research. To recruit participants, the convenience and snowball sampling methods were applied. Research staff first distributed the online survey via social media sites (e.g., TikTok, Instagram, WhatsApp, Facebook). The survey link was also shared via emails and professional networks. This approach allowed for rapid data collection from potential eligible participants who were willing to respond and readily available. After completing the questionnaire, participants were encouraged to share the link within their networks (with friends, colleagues, or contacts who met the inclusion criteria), enabling the sample to expand through peer referrals.

Those who were willing to participate but who did not meet the current study inclusion criteria and those who failed to answer one or all questions or failed to submit their questionnaires were excluded. Eligible participants were requested to fill out a questionnaire sent to them via Google Forms after giving their informed electronic consent. The conditions of anonymity and confidentiality were assured. The questionnaire was developed in local language (Arabic) and took around 10–15 min to complete. The primary section of the questionnaire contained detailed information about the research project. Each participant could complete the survey only once. The ethics approval for the study was granted by the School of Pharmacy at the Lebanese International University. A total of 843 valid responses were received and analyzed.

### 2.2 Sample Size Calculation

The minimum required sample sizes for exploratory (EFA) and confirmatory (CFA) factor analyses were 180 and 360 participants, respectively, following the recommendation of 10 [34] and 20 [35] participants per item of the scale.

### 2.3 Measures

#### 2.3.1 Demographic Characteristics

This part of the questionnaire focused on gathering demographic information about participants, including sex, age, marital status, educational level. We also collected data on personal history of cosmetic procedures for skin (such as chemical peels), personal history of cosmetic surgery for skin (such as a facelift or laser skin resurfacing), and personal history of skin conditions (such as acne, eczema, or psoriasis).

#### 2.3.2 The Skin Dysmorphia Scale (SDS)

The SDS was developed in the Arabic language. The same approach and criteria that were used in the previous study have been adopted for designing widely used and commonly accepted measures in the field, such as the DCQ [30] or the BDD‐D [28]. A first pool of 18 items was developed that align with the Diagnostic and Statistical Manual of Mental Disorders, Fifth Edition, Text Revision (DSM‐5‐TR) criteria for BDD [1]: (1) Preoccupation with appearance, (2) Repetitive behaviors or mental acts to control symptoms, and (3) distress related to symptoms or interference with daily functioning. The first criterion is reflected through items such as “I feel ugly and unattractive because of my skin” or “I am concerned about how my skin changes as I age.” The second criterion was represented in items such as “I wear makeup or creams so that people cannot see my skin,” “I often compare my skin with that of other people” or “I often check my skin in mirrors or other reflecting objects.” The third criterion is assessed by items such as “I feel anxious when I miss one or more days of skin care routine” and “I cancel social activities with family or friends (e.g., going out to dinner, dating, going to the movies, etc.) because of my skin care routine.” Items were designed based on the existing literature on BDD [36, 37], and from the wording used in instruments assessing general or body‐area‐specific BDD symptoms [26, 27, 28, 29, 30, 31, 38]. The original pool of items is presented in Appendix 1. Respondents were asked to rate their concern about their skin's appearance on a five‐point Likert‐type scale ranging from 0 (Never) to 4 (Always). This range of response options was chosen to allow for accurate reflection of respondents' experiences.

#### 2.3.3 The Dysmorphic Concern Questionnaire (DCQ)

This is a short self‐report scale composed of seven items assessing BDD experiences [30]. Each item can be rated on a four‐point scale from 0 (not at all) to 3 (much more than most people). The scale was translated and successfully validated in Arabic, demonstrating good psychometric properties [39]. The DCQ was chosen for the convergent validity assessment because of the similarity of the questionnaire structure with the SDS (Likert‐type item format), and because it is the only measure—to our knowledge—that has been validated in the Arabic language among the general (non‐clinical) population.

#### 2.3.4 The TikTok Addiction Test (TAT)

The TAT is a self‐report tool composed of six items that assess symptoms of TikTok addiction over the past 12 months. The TAT was designed based on the six DSM‐5 criteria of behavioral addictions (salience, tolerance, withdrawal, mood change, conflict, and relapse). Items can be answered on a 5‐point Likert scale ranging from 0 (very rarely) to 4 (very often). In the original validation study, the Arabic‐language TAT showed good psychometric qualities in a multi‐country sample [40]. In the current study, Cronbach's *α* was 0.883.

#### 2.3.5 The Patient Health Questionnaire (PHQ‐4)

This brief tool contains four items measuring anxiety (2 items) and depression (2 items) symptoms over the past 15 days [41]. Items can be answered on a scale varying between 0 (not at all) and 3 (almost every day). Greater scores reflect more severe symptoms. The Arabic‐language version of the scale was utilized [42], which yielded a Cronbach's *α* of 0.87 in our sample.

#### 2.3.6 The Single‐Item Self‐Esteem Scale (SISE)

The SISE consists of a single self‐administered item (“I have high self‐esteem”) that has five answer options ranging from 1 (not at all true of me) to 5 (very true of me) [43]. The version translated, adapted and validated for the Arabic‐speaking context was used [44].

### 2.4 Data Analysis

We conducted the Exploratory Factor Analysis (EFA) and calculated reliability indices using FACTOR 12.04.01 [45], followed by Confirmatory Factor Analysis (CFA) with SPSS AMOS v.30. To evaluate the internal structure of the scale, the full sample was randomly divided into two groups; the EFA was performed on the first group, representing one‐third of the total sample (276 participants), while the CFA was run on the second group (579 participants). Prior to the EFA, data adequacy was assessed with the Kaiser‐Meyer‐Olkin (KMO) measure and Bartlett's test of sphericity. Additionally, item‐level suitability was examined through the Measure of Sampling Adequacy (MSA) [46] and the Anti‐Image Correlation [47]. Items with MSA values below 0.50 were excluded, as such values indicate poor adequacy [46]. The Expected Residual correlation direct Change (EREC) index was further employed to evaluate residual associations between item pairs after accounting for common factors, where values should approximate zero. Strongly correlated item pairs (doublets) were identified and items repeatedly appearing in different doublets were removed [48]. Since the variables were ordinal and several items presented skewness and kurtosis beyond |1|, the EFA was based on a polychoric correlation matrix given the ordinal nature of the variables [49]. The method of estimation was Unweighted Least Squares (ULS) according to the recommendations of the current literature [50]. Factor retention was determined through the Optimal Implementation of Parallel Analysis [51, 52].

To validate the dimensional structure indicated by the EFA, a CFA was conducted on the second subsample using maximum likelihood estimation. Model fit was assessed with several indices, including Standardized Root Mean Squared Residual (SRMR), root mean square error of approximation (RMSEA), Tucker‐Lewis Index (TLI), and comparative fit index (CFI). Adequate model fit was considered for SRMR values ≤ 0.05, RMSEA ≤ 0.08, and CFI and TLI ≥ 0.90 [53].

Furthermore, multi‐group CFA was applied on the full dataset to test measurement invariance across sex [54]. Configural, metric, and scalar invariance were evaluated, with ΔCFI ≤ 0.010 and ΔRMSEA ≤ 0.015 or ΔSRMR ≤ 0.010 serving as evidence of invariance [55, 56]. Group differences in SDS scores were examined with the Mann–Whitney test.

Because multivariate normality was not confirmed, we relied on a non‐parametric bootstrapping approach. Internal consistency was estimated using Cronbach's *α* coefficient and McDonald's ω, while validity evidence was explored through Spearman correlations between the new scale and related constructs (dysmorphic concern, TikTok addiction, depression, anxiety, and self‐esteem).

## 3 Results

Participants had a mean age of 24.39 ± 8.86 and an age range of 18–68 years. Other sample characteristics are shown in Table 1. The most frequently endorsed skin dysmorphia symptom was being concerned about how skin changes with age, which was reported at least rarely by 68.4% of respondents.

**TABLE 1:** Sociodemographic characteristics of the sample (*n* = 843).

Variables	Mean ± SD or *N* (%)
Sex	
Male	197 (23.4%)
Female	646 (76.6%)
Marital status	
Single	677 (80.3%)
Married	166 (19.7%)
Education level	
Secondary or less	151 (17.9%)
University	692 (82.1%)
TikTok addiction (TAT score)	4.97 ± 4.99
Depression (PHQ‐4‐depression)	1.89 ± 1.63
Anxiety (PHQ‐4‐anxiety)	1.75 ± 1.72
Dysmorphic concern (DCQ score)	4.75 ± 4.56
Self‐esteem (SISE score)	3.81 ± 1.02
Skin dysmorphia (SDS score)	7.63 ± 5.57 [min = 0; max = 28]

Abbreviations: DCQ, Dysmorphic Concern Questionnaire; PHQ‐4, Patient Health Questionnaire; SDS, Skin Dysmorphia Scale; SISE, Single‐Item Self‐Esteem Scale; TAT, TikTok Addiction Test.

### 3.1 Factorial Validity

Item relevance was first examined using the MSA index, which showed values above 0.50 for all items, indicating that each contributed to the same underlying construct. Nevertheless, the EREC analysis identified 22 doublets leading to the removal of items 1, 2, 5, 7, 9, 11, 15, 16, 17, and 18, as they were the most frequently represented across the doublets. This refinement left a final pool of seven items. A subsequent factor analysis confirmed the adequacy of the data for EFA, as reflected by the KMO value of 0.873 and a significant Bartlett's test (*p* ≤ 0.001). The results supported a unidimensional factor structure that explained 57.10% of the variance, with satisfactory indices: GFI = 0.993 (> 0.95), UniCo = 0.985 (> 0.95), I‐ECV = 0.886 (> 0.85), and MIREAL = 0.216 (< 0.30). Parallel analysis further corroborated the one‐factor solution.

**FIGURE 1:**
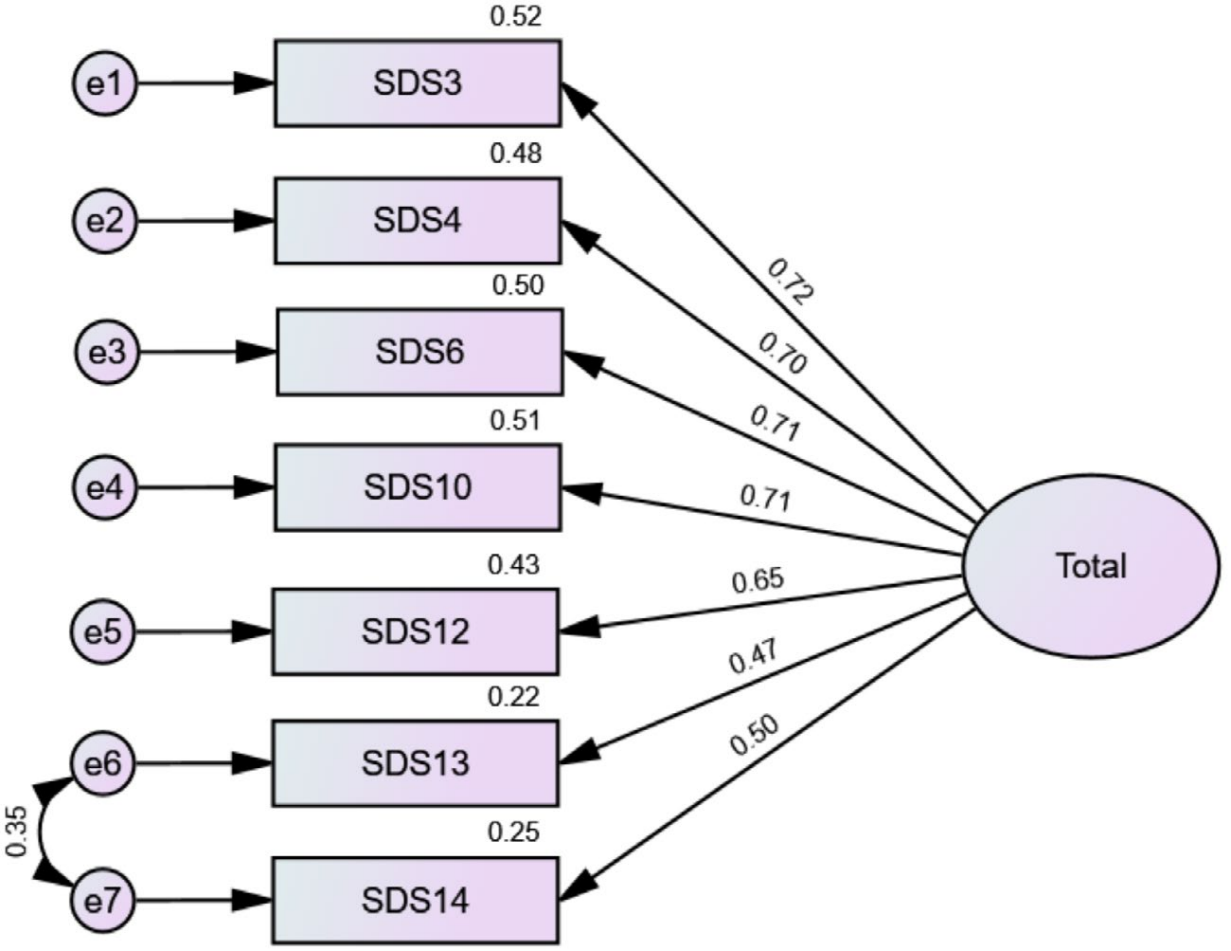
Standardized estimates of factor loadings from the Confirmatory Factor Analysis of the Skin Dysmorphia Scale (SDS).

The unidimensional model was then validated through CFA on the second subsample. Initial model fit was acceptable (SRMR = 0.052, RMSEA = 0.104 [90% CI 0.085–0.123], CFI = 0.932 and TLI = 0.898). However, a high modification index between the residuals of items 13 and 14 suggested the need for a correlated error term. Once this correlation was added, model fit improved substantially (SRMR = 0.026, RMSEA = 0.053 [90% CI 0.032–0.076], CFI = 0.983 and TLI = 0.973) (Figure 1). Internal consistency of the SDS score was good (*α* = 0.83/*ω* = 0.83) [95% CI 0.81; 0.85 for both]. The description of the SDS items can be found in Table 2.

**TABLE 2:** English items of the Skin Dysmorphia Scale, their frequency, and standardized estimates of factor loadings from the Exploratory (EFA) and Confirmatory Factor Analysis (CFA).

Items		Frequency of each item					EFA	CFA
		Never	Rarely	Sometimes	Most of the times	Always		
3	I don't like how my skin looks in photos	278 (33.0%)	231 (27.4%)	228 (27.0%)	85 (10.1%)	21 (2.5%)	0.76	0.72
4	I am concerned about how my skin changes as I age	266 (31.6%)	206 (24.4%)	211 (25.0%)	109 (12.9%)	51 (6.0%)	0.68	0.70
6	I feel ashamed of how my skin looks	545 (64.7%)	151 (17.9%)	108 (12.8%)	32 (3.8%)	7 (0.8%)	0.74	0.71
10	I often wonder whether or not my skin is attractive to other people	435 (51.6%)	189 (22.4%)	149 (17.7%)	46 (5.5%)	24 (2.8%)	0.76	0.71
12	I often feel my skin with my fingers.	282 (33.5%)	214 (25.4%)	207 (24.6%)	91 (10.8%)	49 (5.8%)	0.71	0.65
13	I avoid certain food for a better skin	363 (43.1%)	176 (20.9%)	184 (21.8%)	83 (9.8%)	37 (4.4%)	0.60	0.47
14	I spend more and more time on my skin care routine	324 (38.4%)	200 (23.7%)	197 (23.4%)	84 (10.0%)	38 (4.5%)	0.70	0.50

### 3.2 Sex Invariance

The invariance across sexes was established at the metric and configural, but not at the scalar, levels (Table 3). Higher skin dysmorphia was found in females (Median = 8; IQR = 7) compared to males (Median = 3; IQR = 7), *p* < 0.001, Cohen's d = 0.843.

**TABLE 3:** Measurement invariance of the Skin Dysmorphia Scale score across sex in the total sample.

Model	CFI	RMSEA	SRMR	Model comparison	ΔCFI	ΔRMSEA	ΔSRMR
Males	0.933	0.097	0.049				
Females	0.979	0.057	0.028				
Configural	0.969	0.048	0.049				
Metric	0.968	0.044	0.057	Configural vs. metric	0.001	0.004	0.008
Scalar	0.902	0.071	0.068	Metric vs. scalar	0.066	0.028	0.011

Abbreviations: CFI, comparative fit index; RMSEA, root mean square error of approximation; SRMR, standardized root mean square residual.

### 3.3 Convergent and Concurrent Validity

Higher skin dysmorphia scores were significantly associated with higher general body dysmorphic concerns (rho = 0.46; *p* < 0.001), higher TikTok addiction (rho = 0.29; *p* < 0.001), higher anxiety (rho = 0.35; *p* < 0.001), higher depression (rho = 0.34; *p* < 0.001), and lower self‐esteem (rho = −0.08; *p* = 0.020) (Table 4).

**TABLE 4:** Correlation matrix between continuous variables.

	1	2	3	4	5
1. Skin dysmorphia	1				
2. Dysmorphic concern	0.46***	1			
3. TikTok addiction	0.29***	0.35***	1		
4. Anxiety	0.35***	0.45***	0.32***	1	
5. Depression	0.34***	0.45***	0.32***	0.72***	1
6. Self‐esteem	−0.08*	−0.18***	−0.11**	−0.13***	−0.09**

*Note:* Numbers reflect Spearman correlation coefficients (rho).

* *p* < 0.05.

** *p* < 0.01.

*** *p* < 0.001.

## 4 Discussion

The growing preoccupation with skin appearance and obsession with skincare, referred to as skin dysmorphia, increasingly poses unique challenges to healthcare professionals and thus urgently necessitates a clear, comprehensive approach to assessment and management. The present work is a preliminary endeavor to test the psychometric properties of a new tool designed to identify those with high skin dysmorphia tendencies. The SDS was shown to be valid, reliable, and useful in the evaluation of skin dysmorphia tendency within the community. Although this research constitutes only a preliminary validation of the SDS, it is expected to advance the literature and lay the foundation for future studies on the topic.

EFA identified a unidimensional factor structure for the SDS that was confirmed using CFA in the total sample and in both sex groups. The seven items remaining in the scale had factor loadings for the single factor of 0.47 or higher and resulted in high internal consistency reliability (Cronbach's alpha of 0.83). This is in agreement with previous observations showing that most measurement instruments that have been validated to evaluate BDD symptoms across multiple studies and settings follow a single‐factor structure [32]. The assumption of unidimensionality means that items measure a single underlying latent construct and that item scores can be summed into a single scale score. As a 7‐item self‐report tool, the SDS is brief, quick to administer, and can capture useful data on skin dysmorphia in general population adults.

A positive correlation was found between SDS scores and DCQ scores, thereby supporting the convergent validity of the SDS. The correlation was moderate, indicating a noticeable relationship between skin dysmorphia tendencies and general BDD symptoms that is significant but not overly strong. This can be explained by the fact that the SDS measures concern with a specific area of the body (i.e., the skin) rather than general body dysmorphic concerns. Therefore, our results support that the SDS specifically assesses skin‐related dysmorphic symptoms and holds particular relevance and application over other BDD tools.

Concurrent validity was examined by assessing correlations between SDS scores and scores on other theoretically relevant measures, which are TikTok addiction, depression, anxiety, and self‐esteem. Heavier TikTok users showed significantly higher skin dysmorphia tendencies, which supports that skin dysmorphia symptoms and related behaviors (such as diet trends or skincare routines) seem to be largely influenced by exposure to social media, as attested in previous literature [12, 57]. This concurs with previous suggestions that social media is pervasive in our lives and within today's society, thus amplifying idealized beauty standards and reshaping perceptions of physical attractiveness [58]. Excessive exposure to social media may intensify preoccupation with perceived physical flaws and appearance‐related distress, potentially prompting the seeking of dermatological or surgical interventions [59].

Furthermore, statistically significant correlations were observed between SDS scores and higher depression‐anxiety symptoms. Moreover, skin dysmorphia symptoms were significantly and inversely correlated with self‐esteem levels. Our findings are consistent with previous research in the sense that in the unrealistic pursuit of ageless, flawless skin, individuals end up feeling physically and mentally drained. Intense skincare usage might sensitize the skin and weaken the skin barrier, which can lead in turn to skin damage [22]. Skin‐related appearance concerns can cause low self‐esteem, social withdrawal, depression, and anxiety [9]. For instance, previous research reported that patients with BDD had four‐ to ten‐fold increased likelihood of developing anxiety and depression disorders, respectively [60]. Overall, our results lend support to the close link between skin dysmorphia and psychopathology, which underscores the importance of its early recognition and intervention.

### 4.1 Study Limitations

While this study represents a substantial contribution to the skin dysmorphia literature and has potentially major implications for both research and practice, it also has some limitations. Due to the cross‐sectional design, we could not examine the stability of the SDS over time. Future research repeating application of the SDS at two different times is crucial to explore the scale's test–retest reliability. In addition, only general population adults have been involved in this study. Therefore, the SDS still needs to be psychometrically tested in screening contexts, such as individuals who seek assistance from dermatology or cosmetic surgery settings for dermatological problems relating to their skin. Furthermore, the scale was validated in a Middle‐Eastern and Arabic‐speaking population, making it crucial to investigate validity in populations from other cultural and linguistic backgrounds. Moreover, participants were recruited using an online survey and a combination of a non‐probability convenience and snowball sampling techniques owing to time and resource constraints. This approach carries the risk of selection bias, as the sample may have been biased to include self‐selected individuals interested in mental health and/or biased to exclude individuals who do not have reliable access to the Internet. In this study, this method has attracted mostly female and highly educated participants, which is a typical issue in online surveys. To enhance representativeness in populations beyond those sampled and broaden the generalizability of our results, the use of a randomized sample that involves a more diverse sample is needed for future research. Finally, given the reliance on self‐report measures only and the absence of a clinical evaluation, a specific cutoff for the detection of BDD could not be established in this study. Future studies are warranted to determine the diagnostic sensitivity and specificity of the SDS for skin dysmorphia diagnosis using a physician diagnosis of BDD based either on DSM‐5 diagnostic criteria or on validated structured diagnostic interview for BDD. Defining an empirically‐derived cutoff value will enable us to distinguish between moderate and severe cases of skin dysmorphia and better guide treatment decisions.

### 4.2 Practical Implications and Future Directions

Because of the sensitivity and complexity of skin dysmorphia symptoms, the concerned person can feel ashamed and reluctant to disclose their problem, which can lead to diagnostic and therapeutic wandering. Therefore, the use of a self‐report assessment tool that is reliable, valid, and easy to implement in daily practice is essential. The SDS offers a relatively short, psychometrically sound, and free to use measure that can be utilized in various settings to assess individuals' perspectives on and experiences with their skin appearance concerns. It is our hope that the new scale can be useful in screening individuals for skin dysmorphia and accurately detecting this condition, as this can help dermatologists and surgeons make informed aesthetic treatment and procedure decisions. Before undergoing skin treatments, it is recommended that individuals with aesthetic skin chief complaints be screened for skin dysmorphia to decide whether aesthetic treatments are appropriate and beneficial, or whether the individual should be referred to mental health professionals.

The SDS is not considered to be an official diagnosis of skin dysmorphia, but is rather designed as a screening tool to help mental health professionals (such as a psychiatrist or psychologist) in the diagnostic process. Therefore, dermatologists and cosmetic practitioners are encouraged to include the SDS as part of a thorough psychological assessment of individuals seeking non‐surgical cosmetic procedures or cosmetic surgery for skin improvement prior to administering any of these treatments. Higher scores reflect greater skin dysmorphia and should encourage referral of the person for mental health evaluation. In addition, individuals who undergo cosmetic procedures and report dissatisfaction with the outcomes or experience worsening/no improvement in their mental health condition (i.e., continue to be obsessed/concerned about their skin) after the procedure should also be screened using the SDS to detect and mitigate skin dysmorphia where appropriate. Finally, researchers are invited to conduct future studies on skin dysmorphia using the SDS, which will allow us to understand the magnitude and theoretical underpinning of the condition. Future psychometric studies are needed in different sample groups (e.g., cosmetic skin surgery seekers) to better inform the applicability of the SDS in broad clinical and non‐clinical settings and ensure a comprehensive evaluation of skin dysmorphia across diverse populations.

## 5 Conclusion

Skin dysmorphia can be challenging to detect and manage, as individuals who present with skin‐related appearance concerns or seek skin aesthetic treatment expect clinicians to treat them as having skin issues rather than a psychiatric condition. To fulfill clinicians' needs for informed decision‐making, this study offers the initial validation of a skin dysmorphia measure. Preliminary analyses suggest that the newly developed SDS is a valid and reliable instrument for assessing skin dysmorphic concern and ensuring adequate, timely intervention or referral. The scale can assist clinicians in determining when skin cosmetic procedures may or may not be appropriate, ultimately protecting patient safety and health outcomes. We believe that it is timely and useful that skin dysmorphia be given high priority and be formally recognized by the medical and scientific community, so that affected individuals can get the necessary medical or mental health care. Clinicians and researchers are encouraged to begin using the SDS in their practice.

## Author Contributions

F.F.‐R. and S.H. designed the study; F.F.‐R. drafted the manuscript; S.H. and F.H. carried out the analysis and interpreted the results; F.S. and M.D. collected the data; R.H., M.H., S.O., D.M., and F.H. reviewed the paper for intellectual content; all authors reviewed the final manuscript and gave their consent.

## Funding

The authors have nothing to report.

## Ethics Statement

The ethics approval for the study was granted by the School of Pharmacy at the Lebanese International University. When filling out the online form, each participant provided written informed consent. All methods were performed in accordance with the relevant guidelines and regulations (in accordance with the Declaration of Helsinki).

## Consent

The authors have nothing to report.

## Conflicts of Interest

The authors declare no conflicts of interest.

## Data Availability

Because of ethical committee constraints, none of the data collected or analyzed during this study are publicly available. However, the corresponding author (S.H.) may make the data available upon reasonable request.

## Appendix 1 The Original English‐Language Items of the Skin Dysmorphia Scale (SDS)

**Table:**

	Never	Rarely	Sometimes	Most of the times	Always
1. I think that there are many imperfections in my skin (such as acne scars, blackheads, pimples, dark spots)	0	1	2	3	4
2. I wish my skin would be clearer	0	1	2	3	4
3. **I don't like how my skin looks in photos**	0	1	2	3	4
4. **I am concerned about how my skin changes as I age**	0	1	2	3	4
5. I wear makeup or creams so that people cannot see my skin	0	1	2	3	4
6. **I feel ashamed of how my skin looks**	0	1	2	3	4
7. I am embarrassed when people see my face without makeup or creams.	0	1	2	3	4
8. I feel ugly and unattractive because of my skin	0	1	2	3	4
9. I often compare my skin with that of other people	0	1	2	3	4
10. **I often wonder whether or not my skin is attractive to other people**	0	1	2	3	4
11. I often check my skin in mirrors or other reflecting objects	0	1	2	3	4
12. **I often feel my skin with my fingers**.	0	1	2	3	4
13. **I avoid certain food for a better skin**	0	1	2	3	4
14. **I spend more and more time on my skin care routine**	0	1	2	3	4
15. I spend more and more money on skin care products or skin cosmetic procedures	0	1	2	3	4
16. I cancel social activities with family or friends (e.g., going out to dinner, dating, going to the movies, etc.) because of my skin care routine	0	1	2	3	4
17. I feel anxious when I miss one or more days of skin care routine	0	1	2	3	4
18. I feel depressed or guilty when I miss one or more days of skin care routine	0	1	2	3	4

*Note:* Items in bold are those which were retained after the exploratory factor analysis.
